# Transforming Adult Day Services: Innovative Solutions for Capturing and Improving Health Outcomes

**DOI:** 10.1093/geroni/igaf122.1707

**Published:** 2025-12-31

**Authors:** Tina Sadarangani, Clara Scher, Keith Anderson

## Abstract

Adult day services (ADS) are an essential, but overlooked, part of the long-term care continuum, offering structured activities, social interaction, and health services in a community-based setting. However, the absence of uniform regulations has led to a shortage of standardized data, making it challenging to assess the overall health impact of ADS. This symposium summarizes recent research advancing both how care is delivered in ADS and the ways outcomes are measured–both of which are grounded in community partnered and person-centered approaches that elevate the voices of ADS providers and persons with dementia. The first presentation presents the quality-of-life domains most impacted by ADS according to the perspectives of people with dementia and their caregivers across 4 ADS sites in the U.S. The second presentation describes findings from a feasibility and usability study of CareMobi™, a centralized mhealth platform for family caregivers and ADS to exchange information about a person with dementia’s day-to-day health. The third presentation showcases preliminary findings on health outcomes and healthcare utilization among 1,000 unique ADS participants from 40 ADS providers across 11 U.S. states from the National Adult Day Services Association’s dataset. The fourth presentation summarizes the structures, processes and accomplishments of a learning community developed to implement best practices for dementia-capable ADS as part of a collaboration between 7 ADS sites in California. Discussant remarks will contextualize results with prior research on ADS and highlight implications for future research, policy and practice seeking to amplify the health impact of ADS.

